# Nonbacterial Thrombotic Endocarditis of the Tricuspid Valve in a Male Patient with Antiphospholipid Syndrome

**DOI:** 10.7759/cureus.2695

**Published:** 2018-05-28

**Authors:** Nicole M Yordan-Lopez, Dagmar F Hernandez-Suarez, Lorraine Marshall-Perez, William Marrero-Ortiz, Bladimir Sánchez-Pérez, Angel Lopez-Candales

**Affiliations:** 1 Medicine, University of Puerto Rico School of Medicine, San Juan, PRI; 2 Cardiovascular Medicine Division, University of Puerto Rico School of Medicine, San Juan, PRI

**Keywords:** nonbacterial thrombotic endocarditis, antiphospholipid syndrome, incracardiac mass, myxoma, tricuspid valve, nbte, aps

## Abstract

Valve vegetations in nonbacterial thrombotic endocarditis consist of fibrin and platelet aggregates and can be related to circulating immune complexes, such as in the case of antiphospholipid syndrome. In patients with primary antiphospholipid syndrome, echocardiographic studies have disclosed heart valve abnormalities in about a third of patients. Unusual associations between antiphospholipid syndrome and nonbacterial thrombotic endocarditis include presentation as an intracardiac mass compatible with a myxoma on imaging studies, as well as isolated involvement of the tricuspid valve. Both of these scenarios have been previously reported in female patients. This article presents the case of a 53-year-old Hispanic male with antiphospholipid syndrome who presented to the hospital with symptoms of heart failure and persistent right calf pain. An intracardiac mass attached to the anterior leaflet of the tricuspid valve was found through transthoracic echocardiography. Further imaging studies suggested the mass to be a myxoma and the patient underwent mass excision with tricuspid valve replacement. Pathology report of the surgical specimen was consistent with a diagnosis of nonbacterial thrombotic endocarditis. This case highlights the importance of considering nonbacterial thrombotic endocarditis as a key differential diagnosis in patients with concomitant antiphospholipid syndrome and intracardiac masses, as well as challenges encountered in diagnosis and management.

## Introduction

Nonbacterial thrombotic endocarditis (NBTE) was first described by Zeigler in 1888 when he introduced the term thromboendocarditis to refer to the deposition of fibrin on cardiac valves. It was later called “marantic,” “cachectic,” and “terminal type” endocarditis; in 1936 Gross and Friedberg coined the term “nonbacterial thrombotic endocarditis” [1]. This type of endocarditis is characterized by the presence of vegetations on cardiac valves, which consist of fibrin and platelet aggregates and are devoid of inflammation or bacteria. Its pathogenesis involves endothelial damage and subsequent exposure of the subendothelial connective tissue to the circulating platelets, and can be related to immune complexes, hypoxia, hypercoagulability, and carcinomatosis [2].

Patients with suspected NBTE should be evaluated with doppler echocardiography for the presence of valvular vegetations. In patients with primary antiphospholipid syndrome (APS), echocardiographic studies have disclosed heart valve abnormalities in about a third of patients. Valvular lesions associated with APS can occur as valve masses or thickening, commonly involving the mitral and aortic valves. They are usually described as small and wart-like, varying from pin-head size to 3-4 mm [2, 3]. However, in certain cases, NBTE can be indistinguishable from cardiac neoplasms.

Cardiac valve vegetations masquerading as cardiac myxomas have previously been reported in female patients with APS [4]. Tricuspid valve involvement brings an even greater diagnostic challenge, as right chamber masses have an expansive etiology, with right-sided tumors often being malignant and more aggressive [5, 6]. Though the diagnosis of cardiac tumors relies heavily on the use of multiple imaging techniques, this might prove insufficient; a definitive diagnosis may only be established through histologic examination, which will undoubtedly affect further management decisions [6]. In this report, we discuss the challenges in diagnosis and management of a male patient with APS, who presented with signs of heart failure and a right cardiac mass on imaging.

## Case presentation

A 53-year-old Hispanic male with history of recurrent thromboembolic events while on warfarin anticoagulation therapy presented to the hospital complaining of shortness of breath, abdominal distention, and persistent right calf pain of three-months duration. The patient complained of palpitations, shortness of breath (SOB), and easy fatigability with frequent chest and abdominal pain. Review of symptoms revealed chest pain of four-year duration, a single episode of syncope, and recurrent deep vein thrombosis (DVT) with pulmonary embolisms (PE). The patient had an inferior vena cava (IVC) filter placed two years before due to the recurrence of these thrombotic events despite being on anticoagulation therapy. His only medication was warfarin of unknown dose. He reported being a heavy alcohol drinker and an active smoker with 15 pack a year, but had no history of illicit drug use. Family history was remarkable for mother with type 2 diabetes mellitus, coronary artery disease, and hypothyroidism; and a sister with systemic lupus erythematosus (SLE).

Upon initial examination, vital signs were within normal limits with a temperature of 37.2°C, blood pressure of 140/85 mmHg, and heart rate regular at 80 beats per minute. Body mass index was 25 kg/m^2^. Physical exam was remarkable for a 2/6 holosystolic murmur best heard over the left lower sternal border with a tumor plop, no jugular venous distention (3 cm above sternal notch), hepatomegaly, ascites, bilateral lower extremity hyperpigmentation, and +2 pitting edema. Laboratory studies were pertinent for a normocytic normochromic anemia with hemoglobin of 11.8 g/dl, thrombocytopenia (65,000 x 10^3^/uL), and an international normalised ratio (INR) of 3.83. All other values within complete blood count (CBC) and complete metabolic panel (CMP) were unremarkable. No blood cultures were deemed necessary at the time. After hospital admission and further workup for a hypercoagulable state, levels of cardiolipin Ab IgG, IgM, and IgA were found to be elevated and the patient was diagnosed with antiphospholipid syndrome. Malignancy workup was negative.

**Figure 1:**
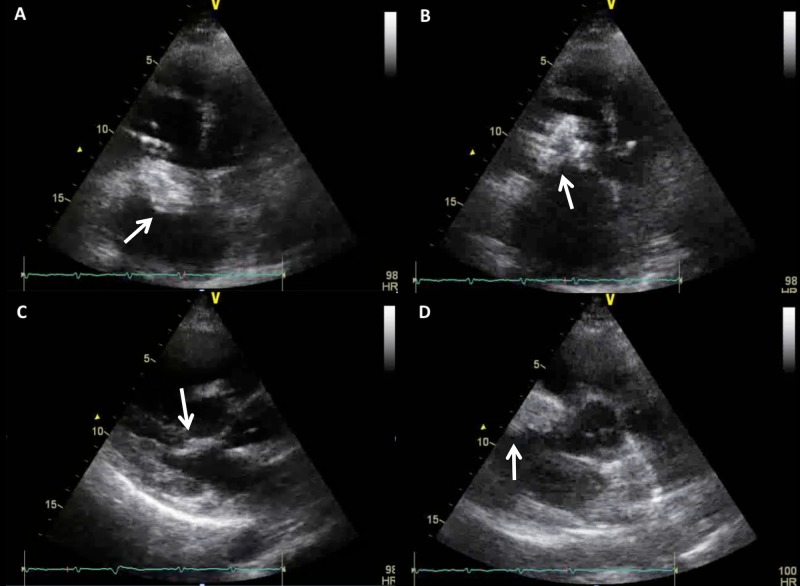
Findings on transthoracic echocardiography. (A-C) A dense echogenic structure, measuring 4.7 cm x 2.7 cm, with areas of calcification was seen attached to the anterior leaflet of the tricuspid valve (TV) and protruding into the right ventricle. (D) No right ventricular outflow tract or pulmonary artery involvement was noted.

**Figure 2:**
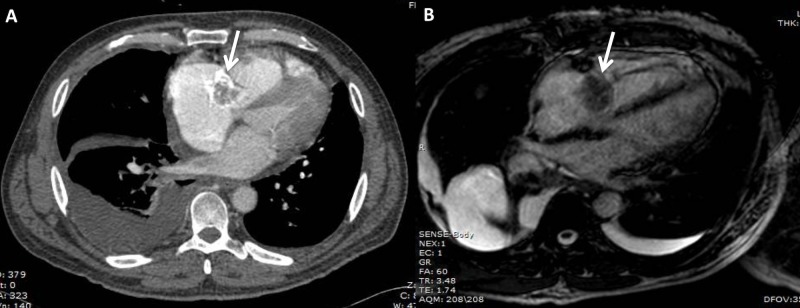
Further imaging with computed tomography (CT) and cardiac magnetic resonance (CMR). (A) Chest CT with a lobulated hypo-attenuating intracavitary right heart mass appearing to be centered in the tricuspid valve. (B) Cardiac magnetic resonance imaging showing no mass-vascularity.

Transthoracic echocardiography (TTE) showed dilated right atria and ventricle with septal flattening and a small pericardial effusion. A dense echogenic, partially calcified structure, measuring 4.7 cm x 2.7 cm, was seen attached to the anterior leaflet of the tricuspid valve (TV) and prolapsing into the right ventricle (Figure 1A-1C). No right ventricular outflow tract or pulmonary artery involvement was noted (Figure 1D). Other pertinent findings included a normal systolic function, and moderate to severe tricuspid regurgitation without evidence of pulmonary arterial hypertension. Further evaluation by two-dimensional transesophageal echocardiography (2D-TEE) confirmed these findings but did not provide additional information. Most likely differential diagnoses of the mass included myxoma vs thrombus. Chest computed tomography (CT) demonstrated a lobulated hypo-attenuating intracavitary right heart mass which appeared to be centered in the tricuspid valve (Figure 2A). Though no vascularity was identified on cardiac magnetic resonance imaging (CMR), findings were suggestive of a cardiac myxoma of the tricuspid valve (Figure 2B).

Together, these findings suggested a right cardiac myxoma causing TV regurgitation and congestion (hepatomegaly, ascites, dilated inferior vena cava). With TV myxoma as the most likely diagnosis, cardiothoracic surgery was consulted for possible surgical excision. Because thromboembolic events could not be definitively attributed to the cardiac mass do to his hypercoagulable state and the patient was believed to be at high risk for surgical complications, the surgical team initially proposed a trial of medical management with anticoagulation and metoprolol before re-evaluation for surgery. However, as the patient's heart failure due to severe TV regurgitation continued to progress, surgical intervention was deemed necessary. Mass resection with TV replacement with a bio-prosthetic valve was performed. Operative findings were pertinent for a destroyed TV with a calcified and adherent soft friable mass at the right lateral atrial wall. Pathology report of the surgical specimen was consistent with chronic endocarditis with thrombi, rejecting the hypothesis of a cardiac myxoma and suggesting a diagnosis of NBTE instead.

## Discussion

Antiphospholipid syndrome has been defined by the development of venous and/or arterial thrombosis, and pregnancy morbidity in the presence of antiphospholipid antibodies (aPL). It can be primary or associated with other diseases, mainly SLE [7]. Antiphospholipid antibodies have been related to higher prevalence of vascular involvement, with multiple studies suggesting that valvular defects can be found in a third of patients with primary APS. Contrarily, NBTE presents more often with thromboembolic events than valvular involvement of mayor hemodynamic significance. Hence, the use of echocardiography has been suggested in patients with aPLs who suffer from embolisms to detect a potential cardiogenic source [3]. Because APS in itself presents an increased risk of pulmonary embolism, it is uncertain whether our patient’s recurrent PEs were a consequence of cardiac thrombi or his underlying hypercoagulable state. Given this patient’s history, we can hypothesize that a thrombus in transit entangled itself in the TV, becoming calcified over time. The mass then led to TV regurgitation and eventual right-sided heart failure with congestive hepatopathy.

Nonbacterial thrombotic endocarditis is usually characterized by sessile, wart-like vegetations that range from pinhead to 3-4 mm in size and most commonly involve the mitral and aortic valves [3]. Although rare, several cases of isolated tricuspid valve involvement related to APS have been reported in the literature [8]. Cardiac masses in patients with APS have proved challenging to physicians, with NBTE vegetations being occasionally misdiagnosed as myxomas due to presenting with similar characteristics on imaging, as well as the possibility of APS and a primary cardiac neoplasm coexisting [4]. Our patient’s presentation of a TV mass was an even greater diagnostic challenge due to the wide etiology of right chamber masses, which although rare, include both malignant and benign neoplasms.

Though primary cardiac tumors have a very low prevalence, the most common primary malignant cardiac tumor, angiosarcoma, has a predilection for the right atrium [5]. Its incidence rate is 0.107 per 1,000,000 person-years [9]. Among the benign cardiac tumors, which account for 90% of primary cardiac neoplasms, myxoma is the most common and is usually located in the atria. However, cardiac metastases are about 20-fold more common than primary cardiac tumors. Finally, thrombi have been found to represent the most frequent cause of cardiac masses [5].

Diagnosis of cardiac masses relies heavily on the use of multiple imaging techniques, including cardiac CT, cardiovascular magnetic resonance (CMR), and echocardiography. While 2D-TTE has been a common imaging technique for the initial evaluation of the heart, a restricted field of view, incomplete ability to evaluate the mass when the body habitus is unfavorable, and poor ability to characterize tissue, give it a disadvantage in the evaluation of cardiac tumors. Conversely, small and very mobile masses, such as vegetations and fibroelastomas, are better appreciated by 2D-TEE than CMR or CT [5]. Three-dimensional (3D) TTE has been suggested to have an advantage over 2D-TTE in imaging cardiac tumors as well as characterizing valve vegetations. Though CMR should be able to identify cardiac masses that do not require excision: pseudotumors, thrombi, lipomas, lipomatous hypertrophy, and papillary fibroelastomas, it lacks an adequate sensitivity for NBTE [5, 6]. When imaging does not suffice, histology of intracardiac masses can be obtained through endomyocardial biopsy, which is mainly indicated for the investigation of right-sided masses showing an infiltrative or obstructive growth pattern, as well as for the differential diagnosis of sarcomas, lymphomas, and metastatic tumors [5].

In this case, initial preoperative 2D TTE suggested a TV myxoma attached to the anterior leaflet, protruding into the right ventricle. Further imaging studies with TEE, chest CT, and CMR appeared to confirm the initial diagnosis. In retrospect, the lack of vascularity in CMR could have been a clue supporting NBTE over myxoma.

Association between APS and NBTE presenting as a myxoma-like mass has been previously described in the literature, but only in female patients [4]. The same has been the case for isolated tricuspid valve NBTE associated with APS [8]. A literature review revealed only scarce reported cases of NBTE presenting as large vegetations in male patients with APS, and those found were restricted to the aortic valve, which is a more common site of NBTE involvement [10]. We found no published studies addressing gender differences in the prevalence of APS with NBTE. However, considering that APS is frequently diagnosed in females and that NBTE is an unusual cause of intracardiac mass, the coexistence of both conditions together in males might be extremely rare. This case highlights the importance of considering NBTE as a key differential diagnosis in patients with concomitant APS and intracardiac masses. Albeit cardiac myxomas are always in the top of the list for primary cardiac masses, in a patient with APS, injury to the endothelium caused by circulating immune complexes along with the hypercoagulable state, should raise suspicion for NBTE instead. In addition, the case reiterates the importance of early use of echocardiography in patients with APS and recurrent emboli despite anticoagulation. This could lead to the detection of a potential cardiogenic source before symptomatic valvular dysfunction.

Our patient was not a candidate for continued medical management due to the severity of his heart failure, as well as an observed lability on warfarin which made it difficult to maintain INR at a therapeutic value. However, recent case reports have shown complete resolution of an aortic valve thrombus with warfarin [10], as well as resolution of TV NBTE in a patient managed with vitamin K antagonists-hydroxychloroquine (VKA-HCQ) [11]. Clinical trials are still necessary to further evaluate said treatment options and develop more robust therapies for NBTE in patients with APS. Currently, surgery is still recommended in those patients presenting with severe valvular regurgitation leading to symptoms of heart failure [4, 8]. Nonetheless, recognizing cardiac masses attributable to NBTE in APS would give a selected group of patients the possibility to undergo a trial of medical management before surgical intervention became necessary.

## Conclusions

In patients with APS with recurrent emboli, the early use of echocardiography can lead to the detection of a potential cardiogenic source before symptomatic valvular dysfunction. In certain cases, NBTE can present as a large mass, poorly distinguishable from cardiac neoplasms in imaging studies. Therefore, though cardiac myxomas are at the top of the list for primary cardiac masses, in patients with APS, NBTE should be considered as a key differential diagnosis. This is especially pertinent as NBTE has been previously reported to respond to medical management, thus certain patients could be prevented from undergoing unnecessary surgical intervention. Surgical management should still be sought in NBTE patients presenting severe valvular regurgitation to prevent progression of heart failure.
